# Developmental expression of membrane type 4-matrix metalloproteinase (Mt4-mmp/Mmp17) in the mouse embryo

**DOI:** 10.1371/journal.pone.0184767

**Published:** 2017-09-19

**Authors:** María José Blanco, Iván Rodríguez-Martín, Ana I. R. Learte, Cristina Clemente, María Gregoria Montalvo, Motoharu Seiki, Alicia G. Arroyo, Cristina Sánchez-Camacho

**Affiliations:** 1 Basic Biomedical Sciences Department, Universidad Europea de Madrid, Villaviciosa de Odón, Madrid, Spain; 2 Centro Nacional de Investigaciones Cardiovasculares Carlos III (CNIC), Madrid, Spain; 3 Doctoral Studies and Research School, Universidad Europea de Madrid, Villaviciosa de Odón, Madrid, Spain; 4 Institute of Medical Science, University of Tokyo, Minato-ku, Tokyo, Japan; Laboratoire de Biologie du Développement de Villefranche-sur-Mer, FRANCE

## Abstract

Matrix metalloproteinases (MMPs) constitute a large group of endoproteases that play important functions during embryonic development, tumor metastasis and angiogenesis by degrading components of the extracellular matrix. Within this family, we focused our study on Mt4-mmp (also called Mmp17) that belongs to a distinct subset that is anchored to the cell surface via a glycosylphosphatidylinositol (GPI) moiety and with the catalytic site exposed to the extracellular space. Information about its function and substrates is very limited to date, and little has been reported on its role in the developing embryo. Here, we report a detailed expression analysis of Mt4-mmp during mouse embryonic development by using a LacZ reporter transgenic mouse line. We showed that Mt4-mmp is detected from early stages of development to postnatal stages following a dynamic and restricted pattern of expression. Mt4-mmp was first detected at E8.5 limited to the intersomitic vascularization, the endocardial endothelium and the dorsal aorta. Mt4-mmp^LacZ/+^ cells were also observed in the neural crest cells, somites, floor plate and notochord at early stages. From E10.5, expression localized in the limb buds and persists during limb development. A strong expression in the brain begins at E12.5 and continues to postnatal stages. Specifically, staining was observed in the olfactory bulb, cerebral cortex, hippocampus, striatum, septum, dorsal thalamus and the spinal cord. In addition, LacZ-positive cells were also detected during eye development, initially at the hyaloid artery and later on located in the lens and the neural retina. Mt4-mmp expression was confirmed by quantitative RT-PCR and western blot analysis in some embryonic tissues. Our data point to distinct functions for this metalloproteinase during embryonic development, particularly during brain formation, angiogenesis and limb development.

## Introduction

Matrix metalloproteinases (MMPs) constitute a large group of endoproteases that are mainly aimed to degrade and modify distinct components of the extracellular matrix (ECM). These enzymes play also a key role as regulators for tumor invasion and vascular formation [1]. The MMPs are mostly secreted, although there is a subgroup that are tethered to the cell membrane (MT-MMPs), either by a single transmembrane domain or by a glycophosphatidyl inositol (GPI) anchor, and with the catalytic site exposed to the extracellular space [2,3]. The membrane anchored MT-MMPs are relevant modifiers of the immediate cellular microenvironment, which modulates cellular functions [4]. This subgroup includes Mt4-mmp, also known as Mmp17, which is a relatively new member of the MMP family and has been poorly characterized to date [5,6]. Mt4-mmp exhibits unique structural and functional characteristics since it has the least degree of sequence identity to the other family members, presenting no shared enzymatic properties with them [1,7]. For instance, its proteolytic activity against ECM proteins is limited, suggesting a certain degree of specificity against its substrates, possibly located in the pericellular space as well as in the plasma membrane associated to lipid rafts [5,6,8].

Mt4-mmp physiological role remains unclear: its loss of function seems to trigger no apparent defects in gestation, growth, morphology, fertility and behavior, and mice showed no apparent abnormal developmental phenotypes [9]. However, it is known that Mt4-mmp is highly expressed in the kidney papilla as well as in the anterior hypothalamus, and null mice have decreased intake of water and daily urine output, suggesting a role for this enzyme in water homeostasis and regulation of the thirst center in mice [10]. The expression pattern of Mt4-mmp appears to be restrained to certain tissues such as the brain, testis, ovaries, leukocytes and colon in humans [2,5,8,11,12], where it may play specific roles [13]. Although the possibility that Mt4-mmp may contribute to development and organogenesis in these tissues seems feasible, likely through the regulation of cell migration, very little is known about its role during development.

Regarding the formation of the brain, the role of Mt4-mmp during the development of the central nervous system (CNS) is not yet clear. It is known that metalloproteinases are generally implicated in neurogenesis, axonal growth and myelin formation, as well as recovery from injury to the nervous system [14]. Indeed Mt4-mmp may be important during brain development, since it is known that it exhibits high levels of expression in the normal brain tissue, being localized in the cerebral cortex and hippocampus at postnatal stages, and the adult hypothalamus [9,10]. It also displays an intense expression pattern in the dorsal cortical plate at late embryonic stages [15–17]. Despite a previous report in zebrafish that shows that its ortholog mmp17b, is required for the proper migration of neural crest cells [17], almost nothing has been reported on the function and expression of membrane type MMPs during embryonic development.

Our previous work has recently revealed a specific function for Mt4-mmp in the proper organization of the aortic wall. Mt4-mmp is detected in the dorsal aorta from early stages of embryonic development, and its loss of function in mice results in the presence of immature vascular smooth muscle cells (VSMCs) and altered ECM, indicating its essential role in arterial vessel wall development and function [18].

It is known that Mt-mmps are also relevantly involved in pathological conditions regulating vascular stability and permeability [1]. Besides, Mt4-mmp has been identified in cancer tissues and previous studies have brought up potential roles for it in cancer progression [4,19–22]. Interestingly, one key aspect of the role of Mt4-mmp in tumor physiology is that it contributes to vessel maturation and stabilization during tumor angiogenesis [1]. This enzyme displays high levels of expression in transformed cells and not in normal epithelium, and it has been shown to display a tumor-promoting activity [4,12,23]. These evidences provide a link between its role in vessel formation and tumorigenesis.

Since to date, little is known about the precise contribution of Mt4-mmp during CNS and blood vessel development, to determine the function of Mt4-mmp during these processes seems paramount to gain further knowledge on its role in pathological phenomena. By using the LacZ reporter under the control of the Mt4-mmp promoter [9] we have detailed that Mt4-mmp is expressed in a specific regionally and timely controlled manner. During early stages of development, Mt4-mmp expression is clearly related to vascular development. Our study reveals that Mt4-mmp is also expressed in distinct brain regions at later stages. Thus, our data suggest important activities of this metalloproteinase during brain formation as well as during vasculogenesis and angiogenesis.

## Material and methods

### Animals

Mutant mice that express LacZ reporter under the control of the endogenous Mt4-mmp promoter were generated by gene-targeting and genotyped as previously described [9]. Mt4-mmp deficient mice were maintained in a C57/Bl6 genetic background. Wild type and Mt4-mmp^LacZ/+^ littermate embryos from pregnant mice were collected between embryonic stages E8.5–18.5 (E0.5 correspond to the day of the vaginal plug) and at postnatal stages P0-P1. Mice were housed in the Centro Nacional de Investigaciones Cardiovasculares Carlos III (CNIC) Animal Facility under pathogen-free conditions and in strict accordance with the institutional guidelines. The protocol was approved by the Committee on the Ethics of Animal Experiments of the CNIC (Permit Number: CNIC-01/13) and the Comunidad Autónoma de Madrid (Permit Number: PROEX 34/13). All animals were sacrificed by cervical dislocation, and all efforts were made to minimize suffering. Animal studies were conformed to directive 2010/63EU and recommendation 2007/526/EC regarding the protection of animals used for experimental and other scientific purposes, enforced in Spanish law under RD1201/2005.

### β-galactosidase staining

Mt4-mmp distribution was determined by using X-gal histochemistry in Mt4-mmp^LacZ/+^ embryos. Small mouse embryos, from E8.5 to E12.5, were fixed by immersion in 0,125% glutaraldehyde in phosphate buffer saline 0.1M pH 7.2 (PBS) for 3h at room temperature and processed for *in toto* LacZ staining according to standard protocols [24]. Briefly, the whole embryo was incubated in X-gal buffer (5mM potassium ferrocyanide, 5mM potassium ferricyanide, 2 mM MgCl_2_, 0.01% deoxycholate acid, 0.02% NP-40 and 0.1% X-gal) at 37°C overnight. Following PBS washing, embryos were fixed in 4% paraformaldehyde (PFA), and then paraffin-embedded and sectioned in the frontal plane at 7 μm thickness. Sections were counterstained with Fast Red to better define distinct embryonic structures. For those embryos older than E12.5 and postnatal stages, animals were transcardially perfused with 0,125% glutaraldehyde in PBS and post-fixed for 2-12h at 4°C. Then, they were cryoprotected in 30% sucrose solution in PBS, embedded in OCT and sectioned in the frontal plane with a cryostat (Leica). Coronal sections of 20-μm thickness were collected and then processed for LacZ staining as described above.

### Protein extraction and western-blot analysis

Protein extracts were obtained from E14.5 wildtype (WT, n = 4), heterozygous (HT, n = 3), and knockout (KO, n = 3) embryonic mouse tissues (forelimb, cerebral cortex and spinal cord), using a cold lysis buffer (20 mM Tris pH 7.5, 2% SDS) and protease inhibitors (Complete, Mini, EDTA-free, Roche). Proteins (30 μg) were resolved by 10% SDS-PAGE in reducing conditions and transferred onto nitrocellulose membranes (0.45 μm, Bio-Rad, Hercules). Membranes were blocked with 5% non-fat milk. Antibodies against polyclonal MMP17 rabbit (EP1270Y clone, ab51075, Abcam), and monoclonal α-tubulin mouse (T6074, SIGMA-Aldrich) were used at 1:1000 and 1:5000 dilution overnight, respectively. Membranes were developed using goat anti-rabbit HRP (1:10000; 111-035-003, Jackson Immunoresearch) and goat anti-mouse HRP (1:10000; 115-035-003, Jackson Immunoresearch) antibodies, recorded by ImageQuant LAS 4000 (GE healthcare Life Sciences) or goat anti-mouse 800 (1:10000; ODYSSEY IRDye®) and recorded by LI-COR Odyssey technology.

### Immunohistochemistry

For immunohistochemical procedures, embryos were fixed in 4% PFA in PBS 0.1M pH 7.2 by immersion or perfusion depending on the developmental stage, cryoprotected in a 30% sucrose solution, and then embedded in OCT and sectioned in the cryostat at 20 μm thickness in the transverse plane. Immunohistochemistry was performed following standard protocols. Primary antibodies used include: polyclonal anti-CD31 hamster (1:1000; MAB1398Z, Millipore), anti-β-galactosidase rabbit (1:1000; ab4761, Abcam), anti-FoxA2 mouse (1:250; F55A10, DSHB), anti-Nkx6.1 mouse (1:1000; 4C7, DSHB), anti-Olig2 rabbit (1:1000; AB9610, Chemicon), anti-ERG-647 rabbit (1:500; ab196149, Abcam) and anti-WT-1 mouse (Wilms´ Tumor-1; 1:50; M3516, Dako). Sections were incubated with the primary antibody diluted in PBS containing 0.1% Triton X-100 and 1% bovine serum albumin (BSA), for 48 h at 4°C. Subsequently, the sections were rinsed in PBS and incubated for 2 hours at room temperature with 488 or 594-Alexa™-conjugated fluorescent antibodies (Molecular Probes, 1:1000). Sections were counterstained with Hoechst (Molecular Probes, 1:1000) for 5 min at room temperature to visualize nuclei.

### RNA purification and quantitative real-time PCR

For RT-PCR analysis, total RNA was extracted from distinct wildtype (WT), heterozygous (HT) and knockout (KO) mouse embryonic tissues at three developmental stages: cerebral cortex, tail, forelimb and heart from E10.5 and E12.5 embryos; cerebral cortex, tail, forelimb, heart, eye, olfactory bulb, spinal cord, mesencephalon and hindbrain from E14.5 embryos (n = 3 for each embryonic stage and genotype). Total RNA was extracted with Tri-Reagent^®^ (Sigma-Aldrich) and purified by using RNeasy Mini Kit (Qiagen Sciences). First-strand cDNA was synthesized with ImProm-II™ Reverse Transcriptase (Promega) according to manufacturer’s instructions. In all cases, a reverse transcriptase negative control was used for testing genomic DNA contamination. Quantitative RT-PCR was conducted with the iQ™ SYBR® Green Supermix (BIO-RAD) on a CFX96 Real-Time System (BIO-RAD), with following conditions: initial denaturation at 95°C for 10 min, followed by 40 cycles of denaturation at 95°C for 15 sec, annealing and extension at 60°C for 1 min. Melt curve analysis from 60°C to 95°C was performed at the end of each run as a quality-control step. The following specific primers were used: for *Mt4-mmp*: 5´-GACCTTCCGTTCCTCAGATG-3´ (F) and 5´-CCTGGTAGTACGGTTGCATG-3´ (R); for *Gapdh*: 5´-AATGCATCCTGCACCACCAA-3´ (F) and 5´-GTGGCAGTGATGGATGGA-3´ (R). Quantitative data were examined using the comparative Ct method [25]. Mt4-mmp expression levels were normalized to the house-keeping GAPDH and presented as a fold increase or decrease relative to the expression levels in the adult cerebral cortex. In all cases, data were plotted as the mean ± standard error of the mean (SEM) derived from duplicate experiments (n ≥ 3).

### Statistics

Statistical differences in protein expression and mRNA levels among different tissues and embryonic stages were assessed by one-way ANOVA followed by post-hoc Student-Newman-Keuls (SNK) multiple comparison test and Student’s t test. Analysis was performed using Graphpad Prism 6.0 and IBM SPSS Statistics v21. Significance was assigned at p<0.05. For Western blot analysis, relative intensity of the bands were quantified by Image J software (https://imagej.nih.gov/ij/) and normalized to α-tubulin ones.

### Data availability

All relevant data are within the paper and its Supporting Information files.

## Results

### Mt4-mmp is expressed at the early embryo and during cardiovascular development

**Fig 1:**
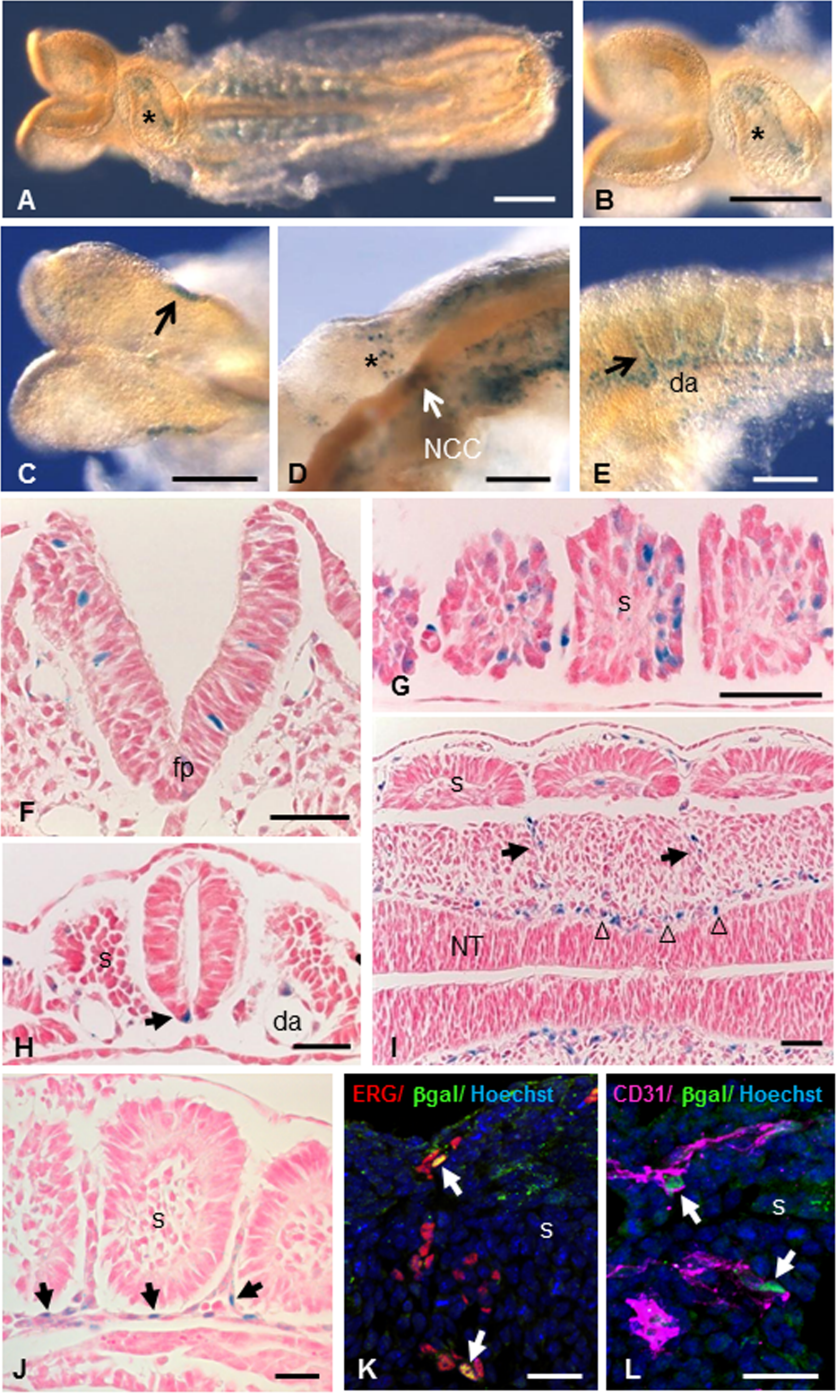
Mt4-mmp expression during early mouse embryonic development. (A-E) Whole-mount β-gal staining in 6–10 somite stage Mt4-mmp^LacZ/+^ embryos. In a ventral view of an 8 somite-stage embryo, it becomes apparent the staining in the endocardial tissue of the primitive heart tube (asterisks in A, B). Staining is observed in the premigratory neural crest cells of a 6 somite-stage embryo (arrow in C). The premigratory and migratory neural crest cells (arrow and asterisk in D) express Mt4-mmp in 10 somite-stage embryos. Somites also show β-gal positive cells (E) in a pattern compatible with intersomitic arteries (arrow in E) beginning to branch from the dorsal aorta. (F-I) β-Gal staining of coronal sections from Mt4-mmp^LacZ/+^ embryos highlights Mt4-mmp distribution in neuroepithelial cells of the neural tube (F), somites (G), floor plate (arrow in H) and dorsal aorta (H) at E8.5 embryos (n = 8). Localization of β-galactosidase was detected in the intersomitic vasculature (arrow in I) and in the perineuronal vascular plexus (arrowheads in I). Detail of LacZ staining in the intersomitic blood vessels (arrows in J). Double-labelled cells for the endothelial markers ERG (red, arrows in K) or CD31 (magenta, arrows in L) and β-gal (green) demonstrate that cells expressing Mt4-mmp in this location are endothelial cells. Abbreviations: da, dorsal aorta; fp, floor plate; s, somite; NCC, neural crest cells; NT, neural tube. Scale bars: 200 μm (A-C), 100 μm (D, E), 50 μm (F-I), 30 μm (J-L).

**Fig 2:**
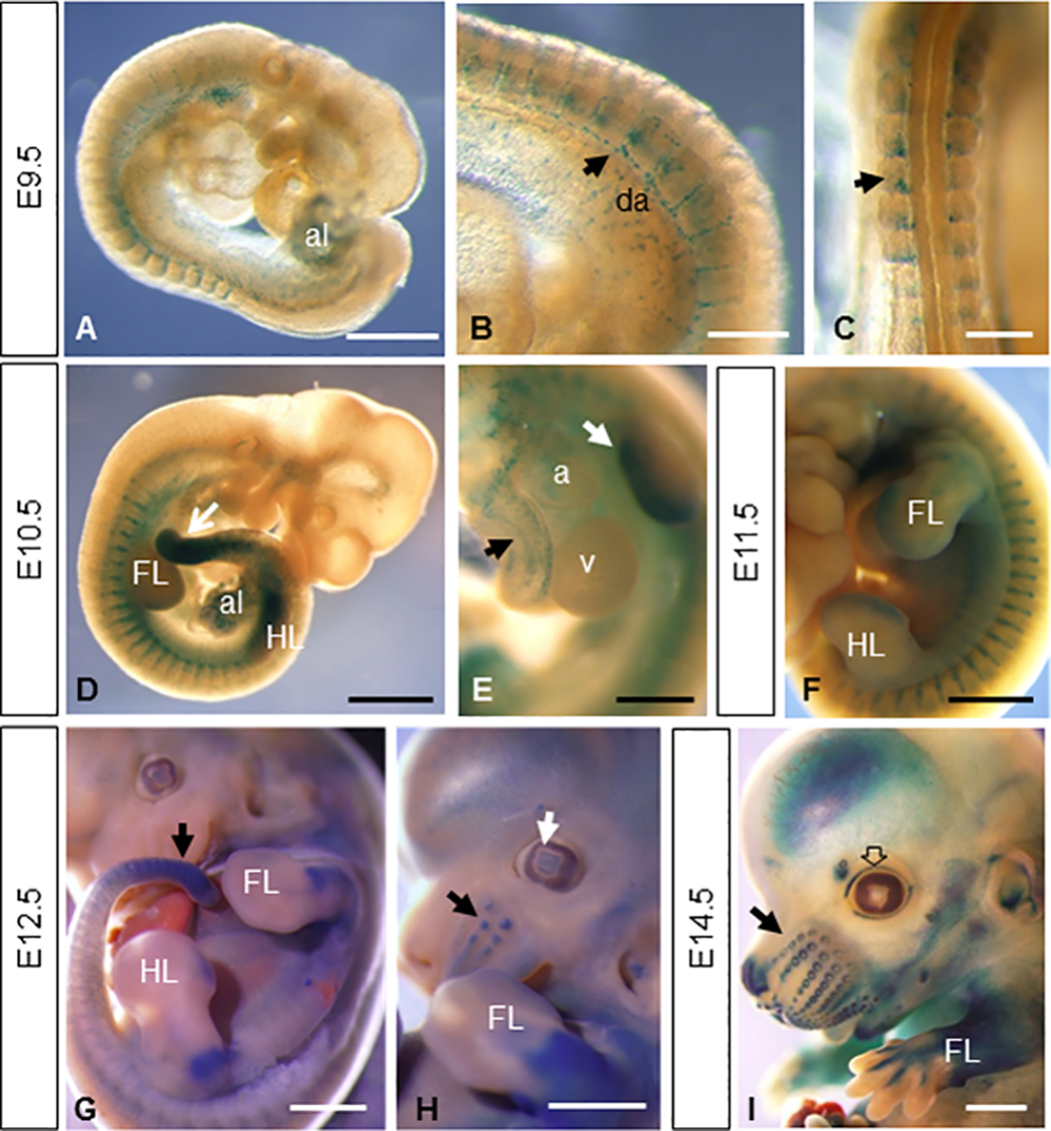
Whole-mount β-gal stained embryos from E9.5 to E14.5. (A-C) E9.5 embryos (n = 7) show reporter expression in the allantois (A), the intersomitic arteries sprouting from the dorsal aorta (arrow in B) and in the posterior portion of the somites (arrow in C). (D-F) This vascular pattern of expression persists during somite development as shown in D and F. Other embryonic tissues like the limb buds, the mesenchymal tail tip (arrow in D and G), the allantois, the atrium and aorta (E) show β-gal staining at E10.5 (n = 5) and E11.5 (n = 3). At later stages of development, expression persists in the limbs (G-I) and appears in the primordium of follicle of vibrissa (arrows in H and I), lens (white arrow in H), eyelid (empty arrow in I), nose, pinna of the ear and brain (n = 6 for E12.5 and E14.5 embryos). Abbreviations: a, atrium; al, allantois; da, dorsal aorta; FL, forelimb; HL hindlimb; v, ventricle. Scale bars: 1 mm (D, F-I), 500 μm (A, E) and 250 μm (B, C).

**Fig 3:**
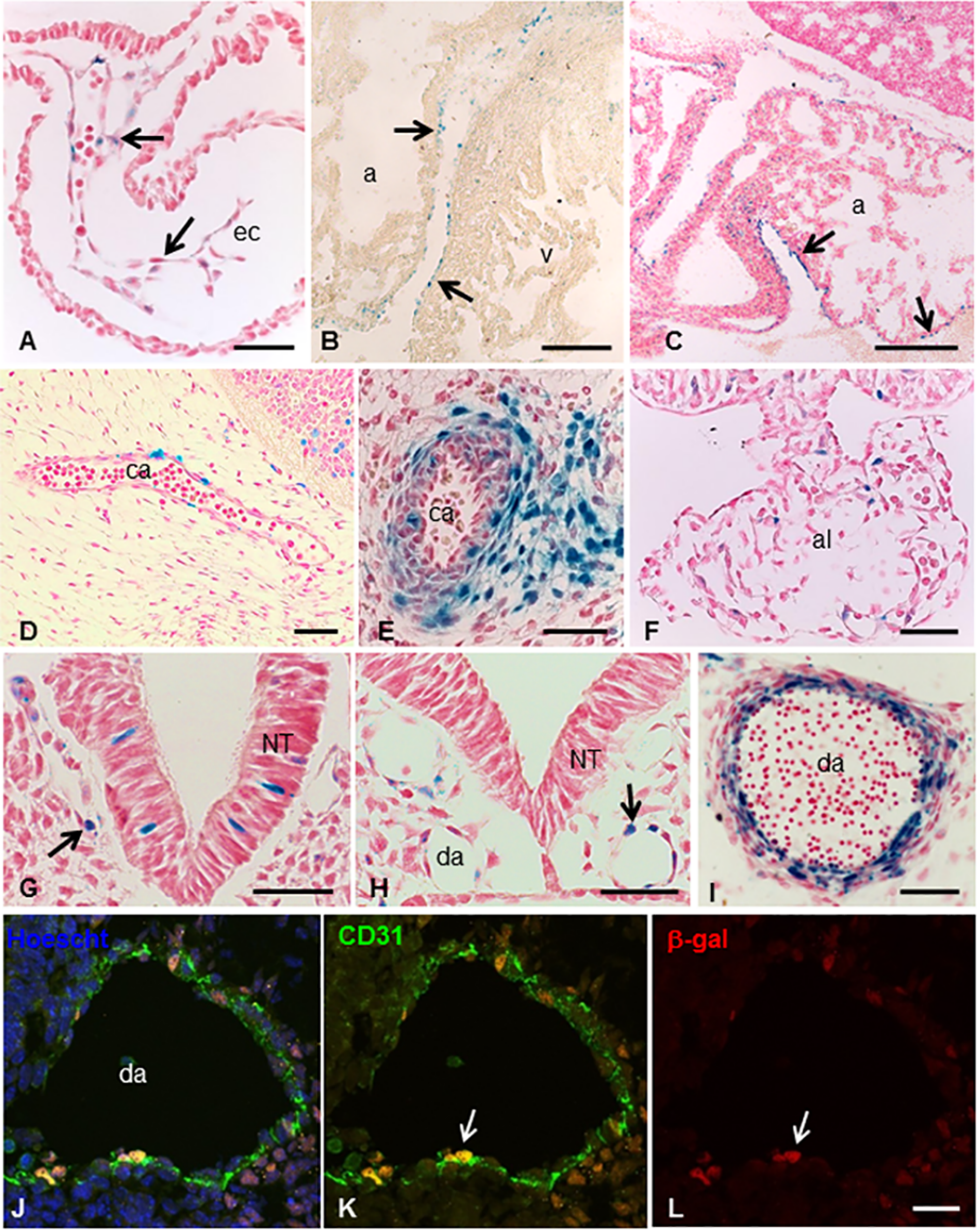
Mt4-mmp^LacZ/+^ expression during cardiovascular development. (A-C) β-gal staining at distinct stages of heart development reveals the expression of Mt4-mmp in the endocardial endothelium of the primitive heart tube at E8.5 (arrows in A) and the epicardium of the atrium and ventricle (arrows in B and C) at E14.5 (B) and E18.5 (C). Expression was also detected in other vascular structures as the branch from internal carotid artery at E11.5 (D) and E14.5 (E) and the allantois at E9.5 (F). By E8.5 β-gal positive cells were detected in the posterior branch of primary head vein (arrow in G) and the dorsal aorta (arrow in H). Labelling in the dorsal aorta persists later in development and by E14.5 Mt4-mmp expression is not only restricted to the endothelium but also located in smooth muscle cells of the aortic wall (I). Double labelling immunohistochemistry for CD31 (J,K) and β-gal (J,L) confirmed the expression of Mt4-mmp in endothelial cells of the dorsal aorta at E10.5 (arrows in K and L). Abbreviations: a, atrium; al, allantois; ca, carotid artery; da, dorsal aorta; ec, endothelial cells; NT, neural tube; v, ventricle. Scale bars: 200 μm (B, C), 50 μm (A, E-H, J-L) and 20 μm (D, I).

The expression pattern of Mt4-mmp was examined at early stages of development by means of β-galactosidase staining using the activity of the LacZ reporter transgene under the endogenous Mt4-mmp promoter in whole mouse heterozygous embryos (Figs 1 and 2) [9]. A consistent staining and distribution of β-gal positive cells was observed among the analysed embryos. LacZ-positive cells were first detected at 4–6 somite embryos (E8.5) in the neural folds corresponding to premigratory neural crest cells as well as in the somites and the mesenchymal presomitic tissue (Fig 1C and 1G). The premigratory and migratory neural crest cells also express Mt4-mmp in 10 somite-stage embryos (Fig 1D). Between 8 and 10 somite embryos, Mt4-mmp expression was primarily associated to the vasculature. Thus, expression was detected in the intersomitic vascularization and the endocardial endothelium of the primitive heart tube (Fig 1A and 1B) and other vascular structures such as the allantois, the umbilical vessels, the vitellin artery and vein and the posterior branch of the primary head vein (Fig 3). Somites also show β-gal positive cells in a pattern compatible with intersomitic arteries beginning to branch from the dorsal aorta (Fig 1E and 1I). In fact, β-gal positive cells are apparent along the entire rostro-caudal extension of the dorsal aorta, particularly in the ventral aortic wall, which possibly correspond to the hemogenic endothelium at these stages. E9.5 embryos showed a similar reporter expression in the intersomitic arteries sprouting from the dorsal aorta and in the posterior and ventral portions of the somites (Fig 1J and Fig 2A–2C). Indeed, double-labelled cells for ERG, an endothelial transcription factor, or CD31 (PECAM-1), an early membrane marker for endothelial cells, and β-gal demonstrate that Mt4-mmp is expressed in these vascular cells. This vascular pattern of expression persists during somite development and begins at the head and the branchial arch vessels. In fact, by E10.5 and E11.5, LacZ staining also localizes mainly in endothelial cells of the dorsal aorta, the branch from the internal carotid artery, and persists in the allantoic vascular plexus (Fig 3D and 3F). By means of double labelling immunohistochemistry for anti-β-gal and the early endothelial marker CD31, we confirmed that Mt4-mmp^LacZ/+^ expression in the dorsal aorta correspond to endothelial cells at E10.5 (Fig 3J–3L). As previously reported, we also found a strong labelling in the dorsal aorta at E14.5 around the whole wall of the vessel (Fig 3I), including endothelial and vascular smooth muscle cells (VSMCs), and persists from E16.5 to E18.5 although the labelling was weaker [18].

Apart from the aorta, expression in VSMCs was also detected in other large blood vessels as the pulmonary or the common carotid arteries at later stages of development (Fig 3E). In addition, β-gal positive cells were disposed surrounding the neural tube, in the perineuronal vascular plexus (PNVP) at E9.5 (Fig 1I). This labelling persists later in development including blood vessel branches from this plexus that enter into the brain parenchyma. Mt4-mmp was also detected during the development of the heart. Thus, Mt4-mmp^LacZ/+^ cells were first detected by E8.5 in the endocardial endothelium of the atrial and ventricular chambers of the primitive heart tube (Figs 1A, 1B and 3A). From E12.5 to E18.5, a similar labeling was found mainly located in the epicardium of the atrium and ventricles (Fig 3B and 3C). We could no detect the expression of Mt4-mmp in the proepicardium nor in the epicardium at earlier stages of development (S1 Fig).

Apart from the cardiovascular system, other embryonic tissues like the mesenchymal tail tip, cells migrating into the branchial arches, premuscle mesodermal condensation/myotome-derived premuscle mass and the rib primordium showed β-gal staining from E10.5 to E12.5 (Fig 2D–2H). As development proceeded, β-gal positive cells were observed in other structures of the embryo such as the cartilage primordium of the body of hyoid bone at E14.5.

**Fig 4:**
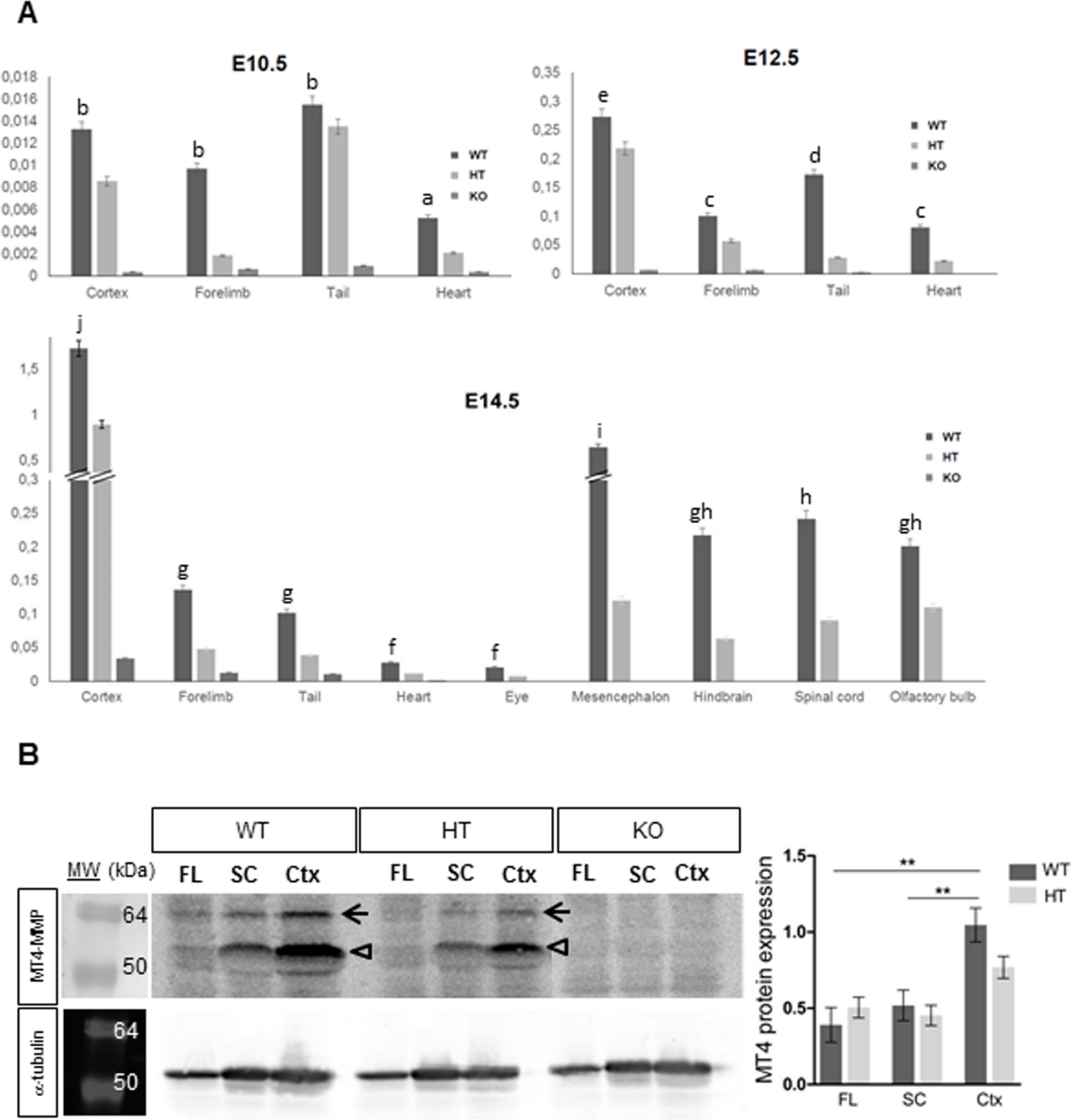
Real-time PCR and western blot analysis of Mt4-mmp expression in mouse embryonic tissues. (A) Real-time PCR analysis was performed using cDNA prepared from E10.5, E12.5 and E14.5 WT, HT and KO mouse embryonic tissues. Mt4-mmp expression increases as development proceeds and particularly, high RNA levels were detected at E14.5 in distinct brain regions as the cerebral cortex, olfactory bulb, mesencephalon, hindbrain and spinal cord. Mt4-mmp RNA levels of were normalized to the housekeeping GAPDH levels and relative to expression levels in the adult cerebral cortex. Data shown are representative of three independent experiments and expressed as the mean ± SEM. In all cases, mRNA levels were significantly reduced in the HT compared to the WT tissues at the distinct embryonic stages analysed. Different letters indicate significant differences (p< 0.05) among distinct tissues of the same embryonic stage by ANOVA and post-hoc analysis (SNK). (B) Total cell lysates from forelimbs (FL), spinal cord (SC) and cerebral cortex (Ctx) of E14.5 WT, HT and KO embryos were analysed by western blotting with the anti-MT4-MMP antibody and anti-α-tubulin antibody for loading control. Specific bands correspond to the precursor of the GPI-anchored form (63 kDa, closed arrow) and the GPI-anchored latent and active forms (55 and 50 kDa, opened arrow) of MT4-MMP. Quantification of Western blot shows significant higher levels of protein expression in the cerebral cortex compared to the spinal cord and the forelimb of WT embryos. MT4-MMP protein levels were significantly decreased in the HT cerebral cortex compared to the WT embryos and were totally absent in all KO embryonic tissues analysed (**, indicates significant differences at p< 0.01).

To confirm our results based on the LacZ reporter expression of Mt4-mmp gene, we performed quantitative RT-PCR analysis in distinct tissues at three developmental stages. Levels of Mt4-mmp expression in the embryonic tissues were always normalized to RNA levels of the enzyme in the adult cerebral cortex according to the high expression reported in this region in previous studies [9,10,15]. Low levels of RNA were detected at E10.5, E12.5 and E14.5 in the tail and the heart of the wild type (WT) embryos, whereas as expected, expression was reduced in the heterozygous (HT) and absent in the knockout (KO) tissues (Fig 4A).

### Dynamic expression of Mt4-mmp during limb development

**Fig 5:**
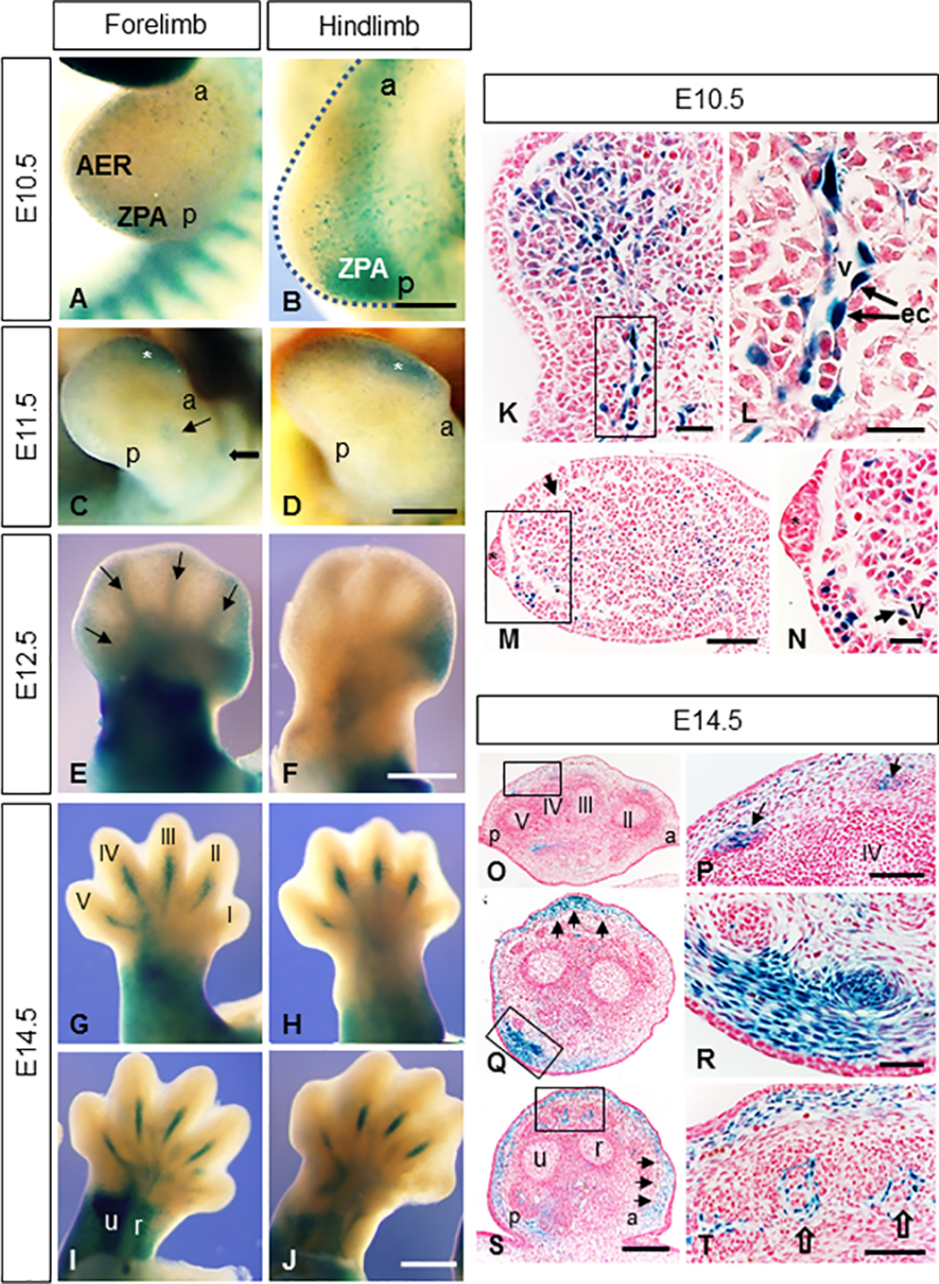
Mt4-mmp^LacZ/+^ distribution during mouse limb development. Mt4-mmp^LacZ+/-^ expression pattern was studied in whole mount limbs at distinct embryonic stages. (A, B) Distal (A) and proximal regions (B) of the limb buds as well the ZPA region show β-gal staining at E10.5. (C, D) Mt4-mmp expression was detected in distal regions of the hand and footplate (asterisks in C,D) as well as in the developing stylopodial and zeugopodial segments (thick and thin arrows in C). (E-J) β-gal staining from E12.5 to E14.5 appears in the stylopodium, zeugopodium and proximal regions of the handplate (E, G-J). In addition, anterior mesenchymal expression is observable in the E12.5 footplate (F). Mt4-mmp expression was first observed in digits at E12.5 (arrows in E) evolving to a pattern restricted to metacarpals and metatarsals of digits II, III, IV and V, either in dorsal (G, H) and ventral side (I, J) at E14.5. (K-N) Paraffin cross sections though hindlimb (K, L) and forelimb (M, N) showed β-gal positive cells in the mesenchyme (arrow in N) and in endothelial cells (ec) of blood vessels entering the limbs (L). The asterisk corresponds to the AER. (O-T) E14.5 limb crossed-sections from distal (O, P) to proximal (Q-T) levels evidence β-gal staining in the tendon blastemas (arrows in P), in subepidermal mesenchymal cells (arrows in Q, S) and in ventral muscle blocks (R) as well as in blood vessels entering the muscles (empty arrows in T). In all sections dorsal is up; ventral is down. Abbreviations: a, anterior; AER, apical ectodermal ridge; ec, endothelial cells; p, posterior; r, radius; u, ulna; v, blood vessel; ZPA, zone of polarizing activity. Scale bars: 500 μm (C-J), 250 μm (A, B, O, Q, S), 100 μm (M, P, T), 50 μm (K, R) and 25 μm (L, N).

Mt4-mmp distribution was also analyzed during mouse limb development showing a very dynamic pattern of expression. Therefore, Mt4-mmp^LacZ/+^ expression was detected by means of β-gal staining during limb development from E10.5 to E16.5 (Fig 5). At early stages of development (E10.5), positive cells were found in distal (forelimb) and proximal (hindlimb) areas as well as in the posterior part of the limbs, particularly in the Zone of Polarizing Activity (ZPA) (Fig 5A and 5B). Paraffin sections at this stage also evidenced positive cells in the mesenchyme (Fig 5K, 5M and 5N) in a pattern compatible with vascular networks established within the limb bud. In addition, endothelial cells were observed in blood vessels entering the hindlimb (Fig 5K and 5L). No expression was detected in the apical ectodermal ridge (AER) by this stage (Fig 5M and 5N). At E11.5, β-gal staining persisted in distal areas of hand and footplate (Fig 5C and 5D). Expression in the zeugopodium and stylopodium (Fig 5C) begins to be observed at this stage, becoming stronger at E12.5. Also, proximal regions of the handplate and developing digits appear strongly stained (Fig 5E). The footplate is less developed than the handplate, showing an interdigital anterior mesenchymal staining (Fig 5F). At E14.5, the stylopodial and zeugopodial regions showed a strong expression, probably related to the establishment of the earliest muscle blocks (Fig 5G–5J). Mt4-mmp expression extended along the metatarsals and metacarpals, either in the dorsal or ventral side (Fig 5E and 5G–5J). This pattern corresponds to the tendon blastemas as it is evidenced by the β-gal positive cells observed in the paraffin crossed-sections though the digits (Fig 5O and 5P). A strong and consistent expression is observed at E14.5 in subepidermal mesenchymal cells and in ventral muscle groups (Fig 5Q, 5R and 5S). Limb sections at the level of proximal radius and ulna, revealed that LacZ expressing cells are related to the developing vascular network irrigating the dorsal muscleskeletal elements (i.e. tendons/ligaments) (Fig 5R, 5S and 5T).

To confirm our results, we performed RT-PCR analysis that revealed Mt4-mmp expression in the WT forelimbs at E10.5, E12.5 and E14.5 whereas RNA levels were reduced in the HT and absent in the KO forelimbs (Fig 4A). As shown by means of β-gal staining, Mt4-mmp RNA levels significantly increase during development (p< 0.05; Fig 4A). In addition, analysis by western-blot confirmed the expression of the protein in the forelimbs of E14.5 WT and HT embryos while it is absent in the KO embryo (Fig 4B).

### Mt4-mmp is highly expressed during brain development

**Fig 6:**
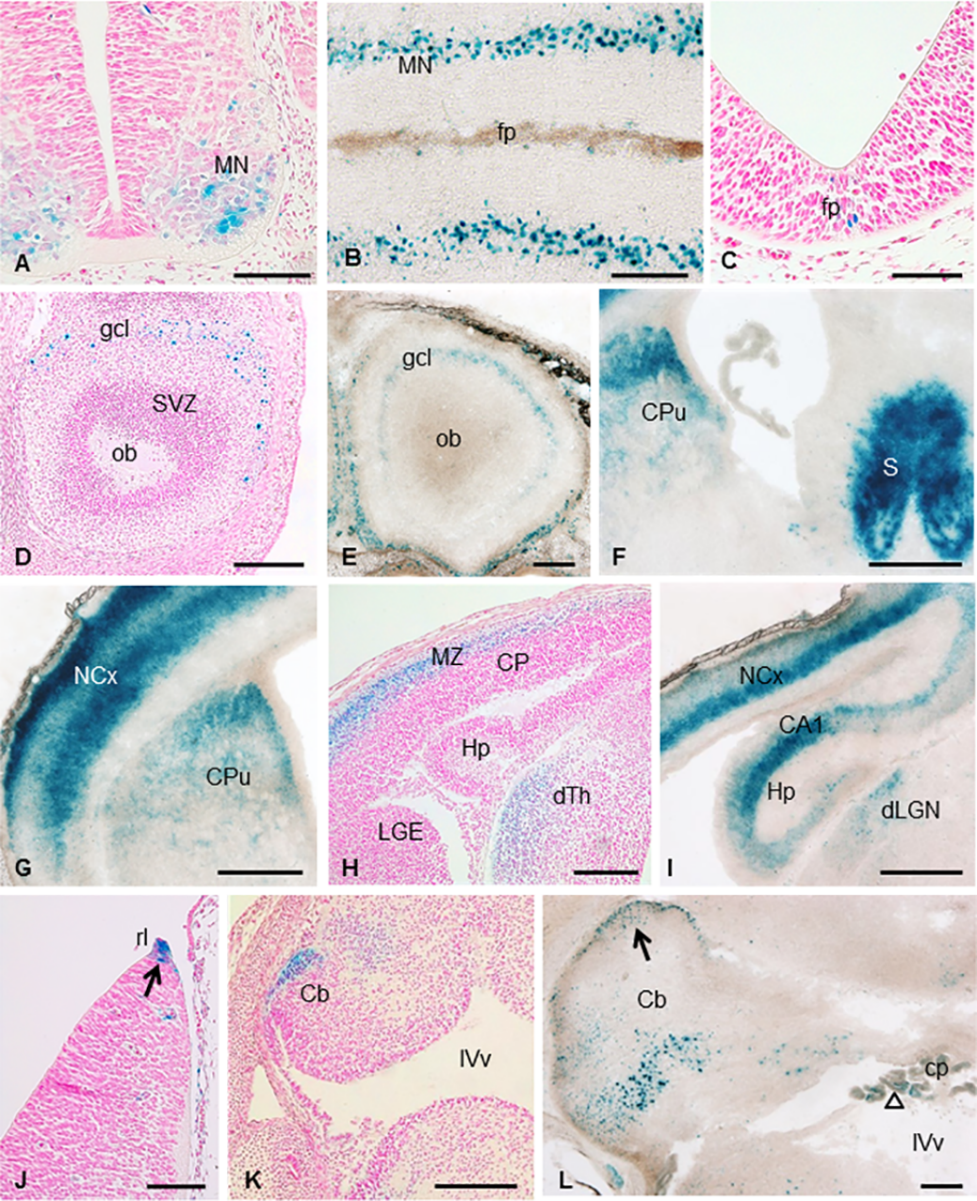
Expression of Mt4-mmp in the developing mouse brain. β-gal staining of coronal sections of embryonic E11.5 (A) and longitudinal sections of E18.5 Mt4-mmp^LacZ/+^ embryos (B) (n = 3) revealed expression of Mt4-mmp in the ventral column of motoneurons in the spinal cord. In addition, β-gal positive cells were detected in the floor plate of the mesencephalon at E11.5 (C). LacZ-positive cells were located in the granular cell layer of the olfactory bulb at embryonic stage E14.5 (D) and postnatal stages (E). Reporter expression was also detected in the septum, striatum, at deep and superficial layers of the cerebral cortex, the hippocampus and the dorsal thalamus and dorsal lateral geniculate nucleus at E14.5 (H) and P1 (F,G,I; n = 3). Mt4-mmp expression was located at the rhombic lip at E12.5 (arrow in J) and the cerebellum primordium at E14.5 (K). By postnatal stages, many β-gal-positive cells were located at the cerebellum possibly corresponding to Purkinje cells and in the external granule layer (arrow in L). At this level, staining was also detected in the choroid plexus of the IV ventricle (empty arrow in L). Abbreviations: Cb, cerebellum; cp, choroid plexus; CP, cortical plate; CPu, caudate putamen; gcl, granular cell layer; dLGN, dorsal lateral geniculate nucleus; dTh, dorsal thalamus; Hp, hippocampus; MZ, mantle zone; NCx, neocortex; ob, olfactory bulb; rl, rhombic lip; S, septum; SVZ, subventricular zone; IVv, IV ventricle. Scale bars: 500 μm (F, G, I), 200 μm (B, D, E, H, K, L) and 100 μm (A, C, J).

Mt4-mmp expression has been previously reported in the brain at late embryonic and postnatal stages [9,10,15]. Here, we demonstrate that Mt4-mmp is located in the nervous system from the early embryo to postnatal stages of development. Thus, Mt4-mmp expression was first detected associated to blood vessels in the neural parenchyma, the notochord, the floor plate and the neural tube at early embryonic stages as shown in transverse sections (Fig 1F, 1H and 1I) (from E9.5 to E11.5). By E11.5, a strong expression was found in the ventral column of motoneurons of the spinal cord (Fig 6A). We could not find phenotypic differences in the distribution of two transcription factors, Nkx6.1 and Olig2, both involved in the ventral patterning of the neural tube, when comparing the percentage of positive cells between the WT and HT embryos, nor when we analysed the KO embryos (S1A–S1C and S1G Fig). Also, we have reported LacZ staining in the floor plate and immunohistochemistry for FoxA2 revealed that the formation of the floor plate is normally specified and developed in the WT, HT and KO embryos (S1D–S1F Fig). Positive cells for LacZ were also observed in the floor plate of the rhombencephalon and mesencephalon at this stage (Fig 6C). By E14.5, LacZ-positive cells were detected in the olfactory bulb (Fig 6D) and the mantle zone of the cerebral cortex (Fig 6H). Notably, expression of Mt4-mmp was found in the spinal cord, particularly stronger at the cervical and lumbar intumescence levels. Other structures in the brain were also labelled at this stage as the dorsal thalamus (Fig 6H), the dorsal hypothalamus, the rhombic lip and the primordium of the cerebellum (Fig 6J and 6K). Mt4-mmp^LacZ/+^ cells were observed in the choroid plexus of the IV ventricle as well as in the blood vessels in the cerebral parenchyma by this stage.

Later in development, the pattern of expression of Mt4-mmp was similar from E18.5 to postnatal stages P0 and P1. Expression restricted to the motoneuron pool in the spinal cord is maintained at E18.5 (Fig 6B). The β-gal staining of coronal sections from postnatal Mt4-mmp^LacZ/+^ mice revealed expression of Mt4-mmp in the granular layer of the olfactory bulb (Fig 6E). A strong reporter expression was also detected in the septum, caudate-putamen (striatum), at deep and superficial layers of the cerebral cortex and the hippocampus (Fig 6F, 6G and 6I). Many β-gal-positive cells were located at the cerebellum possibly corresponding to Purkinje cells and in the external granule layer (Fig 6L). At this level, staining was also detected in the choroid plexus of the IV ventricle (Fig 6L).

Quantitative RT-PCR analysis confirmed increasing RNA levels of Mt4-mmp as brain development proceeds. Thus, expression of the enzyme was low in the cerebral cortex of E10.5 WT embryos but highly increased at E14.5 (significance at p< 0.001) (Fig 4A). At this stage, Mt4-mmp expression was also detected in other brain regions, as the olfactory bulb, spinal cord, mesencephalon and hindbrain. Supporting these data, the expression of the protein MT4-MMP in the brain was confirmed by western blot analysis in the cerebral cortex and the spinal cord of E14.5 WT and HT embryos while no protein levels were detected in the KO. As shown in Fig 4B, the quantity of the enzyme is significantly higher in the cerebral cortex as compared with protein levels detected in the spinal cord and forelimb at this embryonic stage. These results are consistent with our data on the LacZ reporter expression of our gene in these brain regions.

### Mt4-mmp expression in sensory organs and during eye development

**Fig 7:**
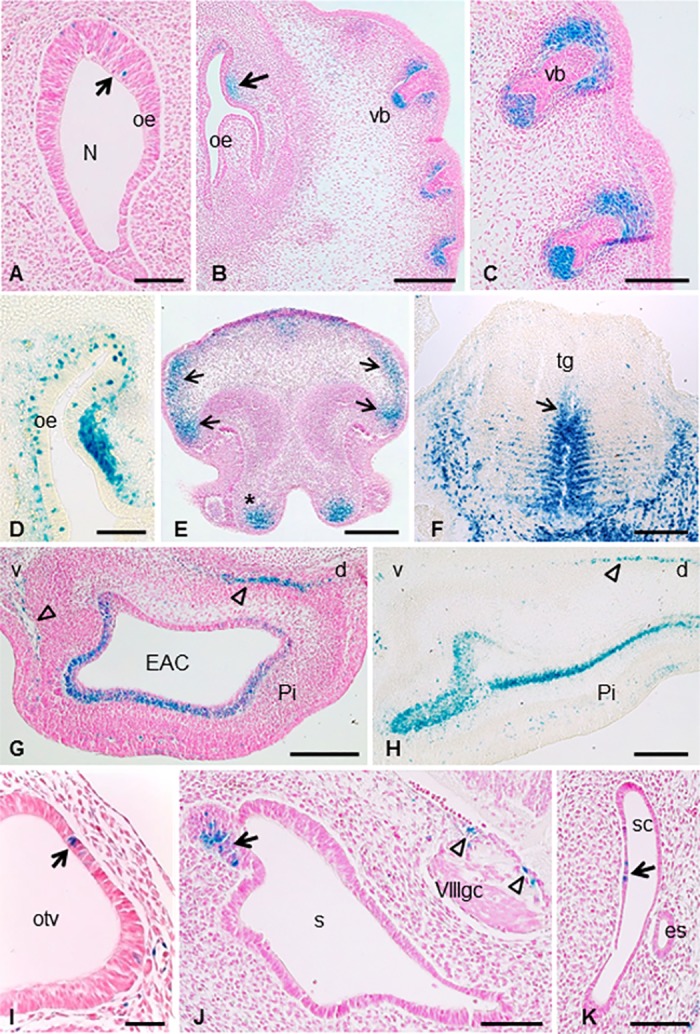
Mt4-mmp expression in sensory organs of the developing mouse embryo. (A-D) Coronal sections through the embryo show the expression of scattered β-gal positive cells dorsally in the olfactory epithelium at the entrance to the nasal cavity at E12.5 (A) and E14.5 (B,D) as well as in the surrounding mesenchyme (arrow in B). High expression of Mt4-mmp was also detected in mesenchymal cells adjacent to the primordium of the follicle of vibrissae (B,C). (E-F) At this embryonic stage, Mt4-mmp expression was also detected in the ventral extremity of the upper jaw (asterisk in E), epithelium of the nose (arrows in E) and median fibrous septum (arrow) and intrinsic muscle of the tongue (F). (G-H) LacZ-positive cells were distributed in the pinna of the ear at E14.5 and E16.5 stages. Empty arrows indicate mesenchymal cells in the pinna. (I-K) From E10.5, the first Mt4-mmp^lacZ/+^ cells were detected in the otic vesicle (arrow in I) and persists at E12.5, in the caudal portion of the saccule (arrow in J), the vestibulocochlear (VIII) ganglion complex (arrowheads in J) as well as in the semicircular canal (arrow in K). Abbreviations: d, dorsal; EAC, external auditory canal; es, endolymphatic sac; N, nasal cavity; oe, olfactory epithelium; otv, otic vesicle; Pi, pinna; s, saccule; sc, semicircular canal; tg, tongue; v, ventral; vb, sensory vibrissae; VIIIgc, vestibulocochlear (VIII) ganglion complex. Scale bars: 200 μm (B, E, F, G, H), 100 μm (A, C, D, J, K) and 50 μm (I).

Mt4-mmp expression was also detected related to the development of distinct sensory organs (Fig 7). Thus, from E12.5 some dispersed LacZ-positive cells were detected dorsally in the olfactory epithelium at the entrance to the primitive nasal cavity (Fig 7A). This expression persists and increases later in development and by E14.5 βgal-positive cells are located in the dorsal part of the olfactory epithelium and the surrounding mesenchyme (Fig 7B and 7D). At E10.5, scattered LacZ-positive cells were found at the otic vesicle (Fig 7I) and this expression is restricted to the caudal portion of the saccule and the associated vestibulocochlear (VIII) ganglia complex at later stages of development (Fig 7J). In addition, some β-gal positive cells were distributed in the semicircular canal from E11.5 embryonic stage (Fig 7K). Sections through the external ear of E14.5 and E16.5 embryos revealed Mt4-mmp labeling in proliferating cells at the base of the developing pinna as well as in mesenchymal cells (Fig 7G and 7H). By E14.5 Mt4-mmp^LacZ/+^ cells were located in the ventral extremity of the lower and upper jaws (Fig 7E) and the epithelium of nose (Figs 2I and 7E). In addition, high levels of expression were found in the mesenchymal layer immediately surrounding the hair follicles primordium of vibrissa and the dermal papilla in the hair bulb (Figs 2H, 2I and 7B and 7C), as well as in the precursor of median fibrous septum and intrinsic muscles of the tongue (Fig 7F) from E12.5 to E16.5.

**Fig 8:**
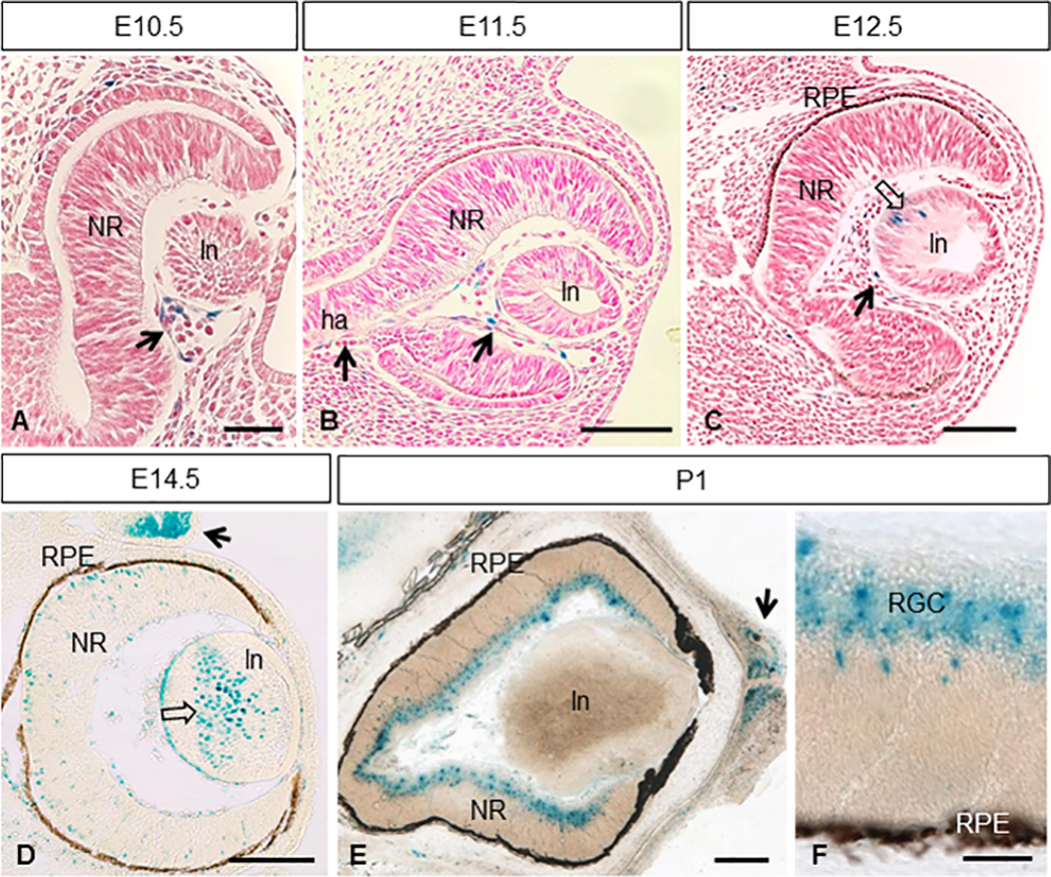
Expression of Mt4-mmp^LacZ/+^ during mouse eye development. β-gal staining of coronal sections through the eye at different stages of development revealed Mt4-mmp expression in the hyaloid artery from E10.5 to E12.5 embryos (arrows in A-C). Scattered LacZ-positive cells were first detected in the lens by E12.5 and persist at E14.5 (opened arrows in C, D). A strong Mt4-mmp expression in the eyelids (arrow in D) and some β-gal positive cells was shown in the neural retina at E14.5 (D). At postnatal stages, expression of Mt4-mmp was distributed particularly in the retinal ganglion cell layer (E, F) of the neural retina and persists in the eyelids (arrow in E). Abbreviations: ha, hyaloid artery; ln, lens; NR, neural retina; RGC, retinal ganglion cells; RPE, retinal pigmented epithelium. Scale bars: 200 μm (D, E), 100 μm (B, C) and 50 μm (A, F).

Finally, we examined Mt4-mmp^Lacz/+^ expression at distinct stages of the eye development. Expression within the optic cup was restricted and first detected by E10.5 in the hyaloid artery (Fig 8A). This pattern of expression in the blood vessels within the eye is maintained throughout development (Fig 8B and 8C) and persists in the central retinal artery entering through the optic disc by E18.5 and postnatal stages (Fig 8E). In addition, cells positive for LacZ were also detected in the lens at E12.5 with a stronger expression at E14.5 (Fig 8C and 8D). Mt4-mmp expression was also observed in scattered cells of the neural retina at E14.5 (Fig 8D). These results are consistent with the detection of the Mt4-mmp RNA levels as demonstrated by RT-PCR in the eye at this embryonic stage (Fig 4A). As development proceeds, the labelling was stronger and mainly restricted to the retinal ganglion cell layer in the postnatal neural retina (Fig 8E and 8F). Notably, a strong expression was also detected in the eyelids from E14.5 to postnatal stages (Figs 2I, 8D and 8E).

## Discussion

The present data reports for the first time that Mt4-mmp expression in the mouse embryo is developmentally controlled and tissue specific, which suggest important roles for this metalloproteinase during brain formation as well as during cardiovascular and limb development.

Previous attempt to analyze Mt4-mmp developmental function were hampered partially due to the lack of apparent abnormal developmental phenotypes in Mt4-mmp null mice, which have been suggestive of redundant roles for this enzyme with other MMPs [9]. To date, only one study has pointed out the requirement of mmp17b, the ortholog of Mmp17 in zebrafish, during cardiovascular development, contributing to vascular smooth muscle cells (VSMCs) in certain aortic regions [17]. Supporting these data, we have recently shown that the activity of this enzyme is essential for arterial vessel wall development and function in adult mice. Thereby, the lack of Mt4-mmp results in the presence of immature VSMCs and altered ECM in the vessel wall leading to a dilative arterial disorder and hypotension. Maturation of VSMCs during aortic wall development involves the c-Jun N-terminal kinase (JNK) intracellular signaling pathway [18]. Notably, the Wnt/planar cell polarity (PCP) pathway involves downstream activation of JNK in the embryo to mediate cytoskeletal rearrangement and cell movement. Our present data prove Mt4-mmp expression in the dorsal aorta and in premigratory and migratory neural crest cells at early stages of development. Thereby, it is possible that Mt4-mmp participates during cell migration in the mouse embryo in a similar manner as described in zebrafish [17].

Expression of MT4-MMP was initially reported in several adult human tissues such as the brain, leukocytes, colon, ovary, testis [5] and the amniochorion [26]. Similarly in mice, Mt4-mmp was detected in distinct organs such as the lung, uterus, spleen, stomach, intestine, testis, ovary, liver, kidney, skeletal muscle and heart [9]. Also high Mt4-mmp expression was described in the cerebrum, particularly in the dorsal cortical plate at late mouse embryonic stages, and the cerebral cortex, hippocampus, caudate-putamen and hypothalamus in the postnatal brain [9,10,15]. However, no detailed expression analysis for this enzyme has been published during early CNS development before. Our study confirms previous data and reports for the first time the detection of Mt4-mmp from early stages of the embryonic brain. As development proceeds, the enzyme localized in other structures, such as the olfactory bulb, mantle zone of the cerebral cortex, septum, hippocampus or the striatum. Even though the role of Mt4-mmp in the brain is not yet clear, our results hint for important functions of its activity during CNS development. Expression from early embryonic stages may involve the activity of the enzyme in neural proliferation and migration, whereas later in development Mt4-mmp may participate in other functions such as synaptic transmission and plasticity due to its expression in the cerebral cortex and hippocampus. Despite to date only one study has proposed a physiological role for Mt4-mmp in the CNS [9], we hypothesize that Mt4-mmp may contribute to these functions by remodeling the ECM locally or interacting with other cell signaling pathways.

We have also shown that, during eye development, Mt4-mmp expression is initially restricted to blood vessels of the hyaloid artery at early stages and it can also be detected in the lens and retinal ganglion cell (RGC) layer in later stages. Supporting our data, it has been reported that Mt4-mmp is selectively expressed in starburst amacrines and in a subset of ON-OFF direction-selective RGCs at postnatal stages, particularly in those RGCs that prefer nasal motion [27]. Moreover, we found that Mt4-mmp is expressed in the dorsal thalamus by E14.5 and in the dorsal lateral geniculate nucleus postnatally, one of the major central targets for retinal axons. These data suggest the involvement of this protease during axon guidance of visual axons. In fact, there are some evidence for the requirement for MMPs in the development of the retinotectal projections. Thus, broad inhibition of MMP activity in *in vivo* exposed preparations of *Xenopus* brain resulted in misguidance of RGC axons at the level of the optic chiasm and the optic tectum [28,29]. Furthermore, it was demonstrated that Mmp14a, one paralog of another membrane-anchored MMP (Mmp14/Mt1-mmp), is necessary for retinal neurogenesis and differentiation and RGC axon innervation of the optic tectum in zebrafish larvae [30]. Also another transmembrane MMP, Mt5-mmp, which is highly expressed in the hippocampus and cerebellum, has been directly involved in axon growth and detected at the growth cone [31,32]. Nevertheless, it remains to be determined if Mt4-mmp also participates in axon guidance and growth of retinal projections and if it is involved during the proliferation and maturation of the retina.

As shown in our study, Mt4-mmp is highly and dynamically expressed during the formation of the mouse embryonic limb since early developmental stages, being detected in the zone of polarizing activity (ZPA) and the mesenchyme underlying the apical ectodermal ridge (AER), respectively. Notably, cells in the ZPA secrete the morphogen Sonic Hedgehog (Shh) which regulates digit fate and is crucial to establish the anterior-posterior skeletal pattern of the limb at these stages [33,34]. In the proximo-distal axis, outgrowth of digits requires fibroblast growth factor (FGF) signaling emanating from the AER [35]. Since the appropriate limb development requires the coordination of both signaling pathways in the anterior-posterior and proximo-distal axis, it is likely that Mt4-mmp associates with both. Regarding this idea, a recent study has pointed out that the activity of the membrane-anchored metalloproteinase-regulator RECK is essential for limb development, directing the expression of genes in three signaling centers essential for limb growth and patterning: DE (producing Wnt7a), ZPA (producing Shh), and AER (producing Fgfs) [36]. Whether the expression of these morphogens is altered in the Mt4-mmp mutant embryonic limb has not been addressed so far, although limb development seems to be normal in the mutant embryo.

In addition to the latter, our data shows Mt4-mmp expression in ZPA cells at E10.5, suggesting that Mt4-mmp might be related to the role proposed for Shh on the anterior-posterior organization of muscles acting through migrating progenitors coming from the somites. These cells, once in the limb bud, will respond to Shh signal from the posterior part of the limb [37]. As limb development proceeds and the skeletal elements differentiate, Mt4-mmp is expressed in the tendons of digits and in developing muscle blocks in the stylopodium and zeugopodium confirming previous evidence [38–40]. Moreover, limb expression of Mt4-mmp between E12.5 and E14.5 mimics that of Scleraxis (Scx), a highly specific marker for tendons [41].

We have also demonstrated that the expression of Mt4-mmp is linked to the vascularization of the limb buds between E10.5-E11.5. At these stages, angiogenesis initiates as sprouts from the dorsal aorta, invading the limb and forming a vascular plexus within the limb mesenchymal core [42]. In addition, concomitant vasculogenesis occurs by migration of somite-derived angioblasts to be integrated into the developing vascular plexus [43]. Both events can be recognized in the limbs included in this study. Thus, β-gal positive cells are found in the mesenchyme extending along the network of microvessels throughout the limb. Also, Mt4-mmp was expressed in the blood vessels entering the limb buds at E10.5 in a pattern compatible with that reported by [44]. Regarding limb development, Mt4-mmp expression also seems to be related to muscle progenitors in the embryonic limb bud by E10.5. Thus, Mt4-mmp expression is observed in cells at the base of the hindlimb, compatible with migrating myogenic and angioblastic cells as described by [45]. Indeed, expression of the enzyme is associated to the initial formation of blood vessels in other structures and organs of the embryo, such as the eye, heart or the nervous system. From our data, Mt4-mmp expression is also located in distinct blood vessels (carotid artery, umbilical vein, vitellin artery an vein, primary head vein, PNVP) at early stages, when the first vascular system start to develop. Mt4-mmp expression in the dorsal aorta has been reported as early as E10.5 in periaortic progenitors and in the adult mouse [10,18], but here we report that Mt4-mmp is detected even earlier in development (from E8.5), possibly related to the hemogenic endothelium. Altogether, our data reveal that Mt4-mmp is located in endothelial cells (EC) of blood vessels of distinct organs at early embryonic development, which contrasts with the fact that its expression seems to be downregulated in EC of more mature blood vessels [9]. The role of Mt4-mmp in blood vessel formation during embryogenesis remains unknown, since only in pathological conditions, Mt4-mmp has been involved in angiogenesis by contributing to vessel maturation and stabilization during tumor progression [1]. For instance, in breast cancer, MT4-MMP induces tumor growth and metastasis by stimulating angiogenesis in the tumor [23,46]. Notably, this angiogenic response requires its proteolytic activity in the tumor compartment but not from the host-derived stroma [46]. Thus, MT4-MMP expression in host cells does not affect angiogenesis. Therefore, it is conceivable that as occurs during metastatic dissemination, Mt4-mmp also contributes to create a permissive microenvironment that favors cell migration in the embryo.

Regarding the proteolytic activity of Mt4-mmp, only few substrates have been identified so far, including pro-TNFα, α-macroglobulin [47], low density lipoprotein receptor related protein (LRP) [48] and aggrecanase (ADAMTS-4) [49] Recently, the matricellular glycoprotein osteopontin (OPN) has been also confirmed as another substrate cleaved by Mt4-mmp and essential during the formation of the aortic wall [18]. Interestingly, OPN is expressed in other tissues during embryonic development where Mt4-mmp also localizes, as the kidney, cartilage and brain. Thus, OPN is expressed in several neural nuclei of the developing rat brainstem and cerebellum [50] as well as in active macrophages in the embryonic and early rat postnatal brain [51]. Previous data suggest that OPN may contribute to the migration and phagocytic function of brain macrophages in the developing rat brain. In fact, OPN functions as a potent chemoattractant that promotes the migration of cells of monocyte/macrophage lineage [52]. However, if OPN has a specific function dependent on Mt4-mmp proteolytic activity during embryogenesis as in the aortic development, remains unknown. It should be mentioned that apart from its proteolytic function, Mt4-mmp is also able to modify intracellular response through the activation of the growth receptor EGFR. Thus, Mt4-mmp positively promotes cancer cell proliferation by enhancing the EGFR signaling pathway independently on the catalytic site of the enzyme [53].

In summary, in this study we reported the spatiotemporal expression of Mt4-mmp during mouse embryonic development. The present data reveals that this GPI-anchored metalloproteinase is expressed in a dynamic pattern of expression from early stages of development to postnatal stages with a high expression of this enzyme during vascular development and brain formation. Our results point for key functions of Mt4-mmp during the CNS and limb development as well as in angiogenesis. If similarly to cancer cells, non-catalytic and catalytic functions of Mt4-mmp can be required for proliferation and for angiogenesis and cell migration in the embryo requires further research. In these processes, both ECM remodeling at pericellular level and activation of distinct intracellular signaling pathways to direct cell movement are crucial events that may involve Mt4-mmp activity.

## Supporting information

S1 Fig Mt4-mmp is not expressed in the proepicardium and epicardium at early developmental stages. (A-B) LacZ staining of sagital sections from E9.5 Mt4-mmp^LacZ/+^ embryos illustrating the lack of βgal-positive cells in the region of the proepicardium. (C-H) Double labelling immunohistochemistry for anti-WT-1 (arrows in red) and anti-βgal (green). No doubled-positive cells were detected in the region of the epicardium nor in the proepicardium of E10.5 heterozygous embryos. Abbreviations: EC, epicardium; PE, proepicardium; V, ventricle. Scale bars = 50 μm (A,B); 40 μm (C-H).
(TIF)

S2 Fig Ventral patterning and specification of the embryonic neural tube. (A-C) Double-labelling immunohistochemistry for the transcription factors Nkx6.1 (red) and Olig2 (green) in the neural tube of E10.5 WT, HT and KO embryos. (D-F) Immunostaining for FoxA2 (red) demonstrated that the floor plate is properly specified in the WT, HT and KO neural tubes. Sections were incubated with Hoechst for nuclear staining (blue). (F) Quantification of the percentage of Nkx6.1 and Olig2-positive cells relative to the total area of the neural tube in the WT, HT and KO embryos. Data are presented as mean ± SD (n = 3 per each genotype). One-way ANOVA analysis revealed no statistical differences in the percentage of positive cells among the WT, HT and KO neural tubes. Abbreviations: fp, floor plate. Scale bars: 50 μm (A-C); 20 μm (D-F).
(TIF)

S3 Fig Database containing quantified data from real-time PCR and western blot analysis (in Fig 4) and ventral neural tube patterning (in S2 Fig). (XLSX)
