# Supplementary figures and images for: Developmental expression of membrane type 4-matrix metalloproteinase (Mt4-mmp/Mmp17) in the mouse embryo
Source: PLoS One. 2017 Sep 19;12(9):e0184767. doi: 10.1371/journal.pone.0184767 (PMC5604975; doi:10.1371/journal.pone.0184767)

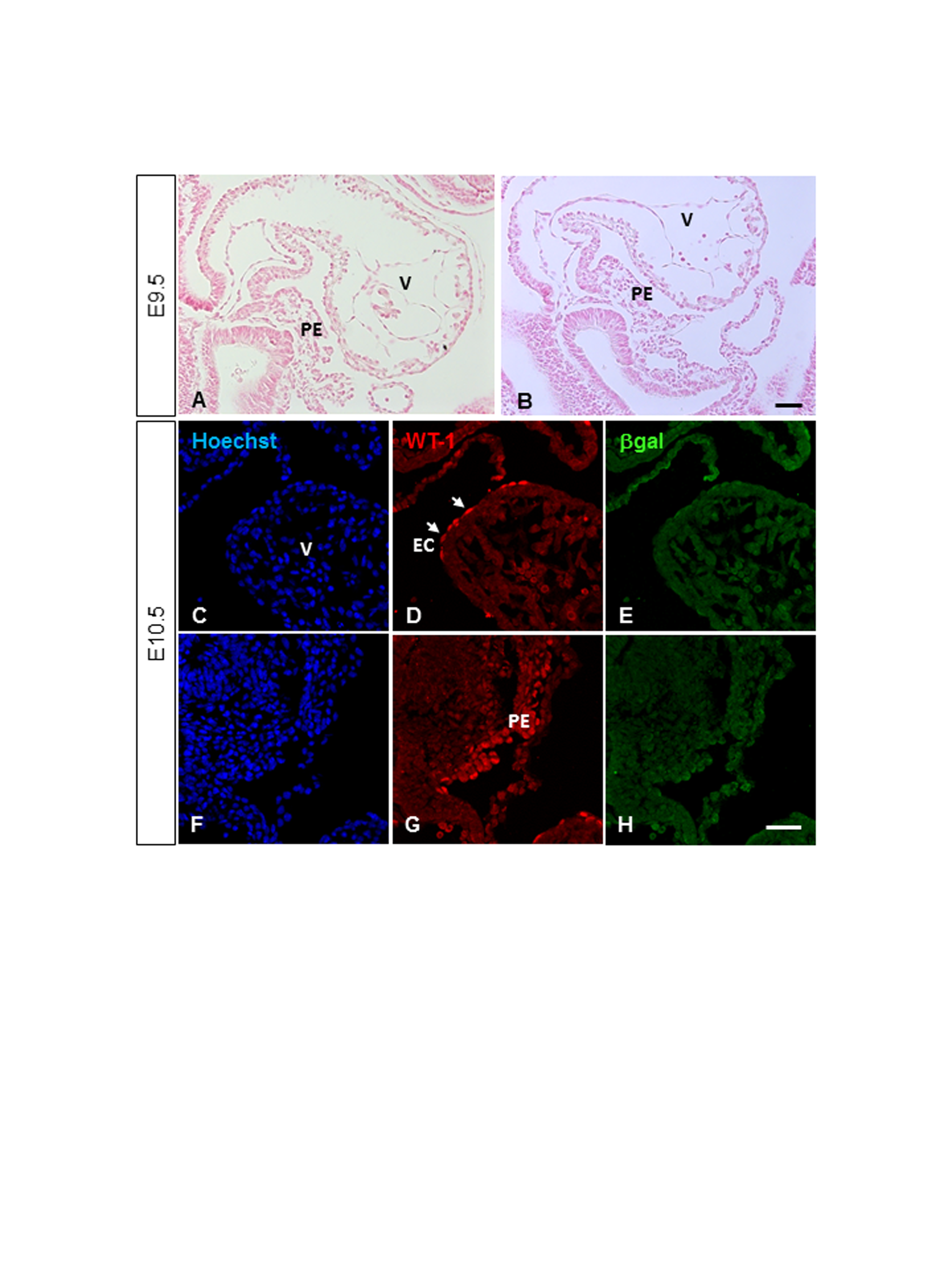

Supplement: S1 Fig — (A-B) LacZ staining of sagital sections from E9.5 Mt4-mmpLacZ/+ embryos illustrating the lack of βgal-positive cells in the region of the proepicardium. (C-H) Double labelling immunohistochemistry for anti-WT-1 (arrows in red) and anti-βgal (green). No doubled-positive cells were detected in the region of the epicardium nor in the proepicardium of E10.5 heterozygous embryos. Abbreviations: EC, epicardium; PE, proepicardium; V, ventricle. Scale bars = 50 μm (A,B); 40 μm (C-H). (TIF) [file pone.0184767.s001.tif]

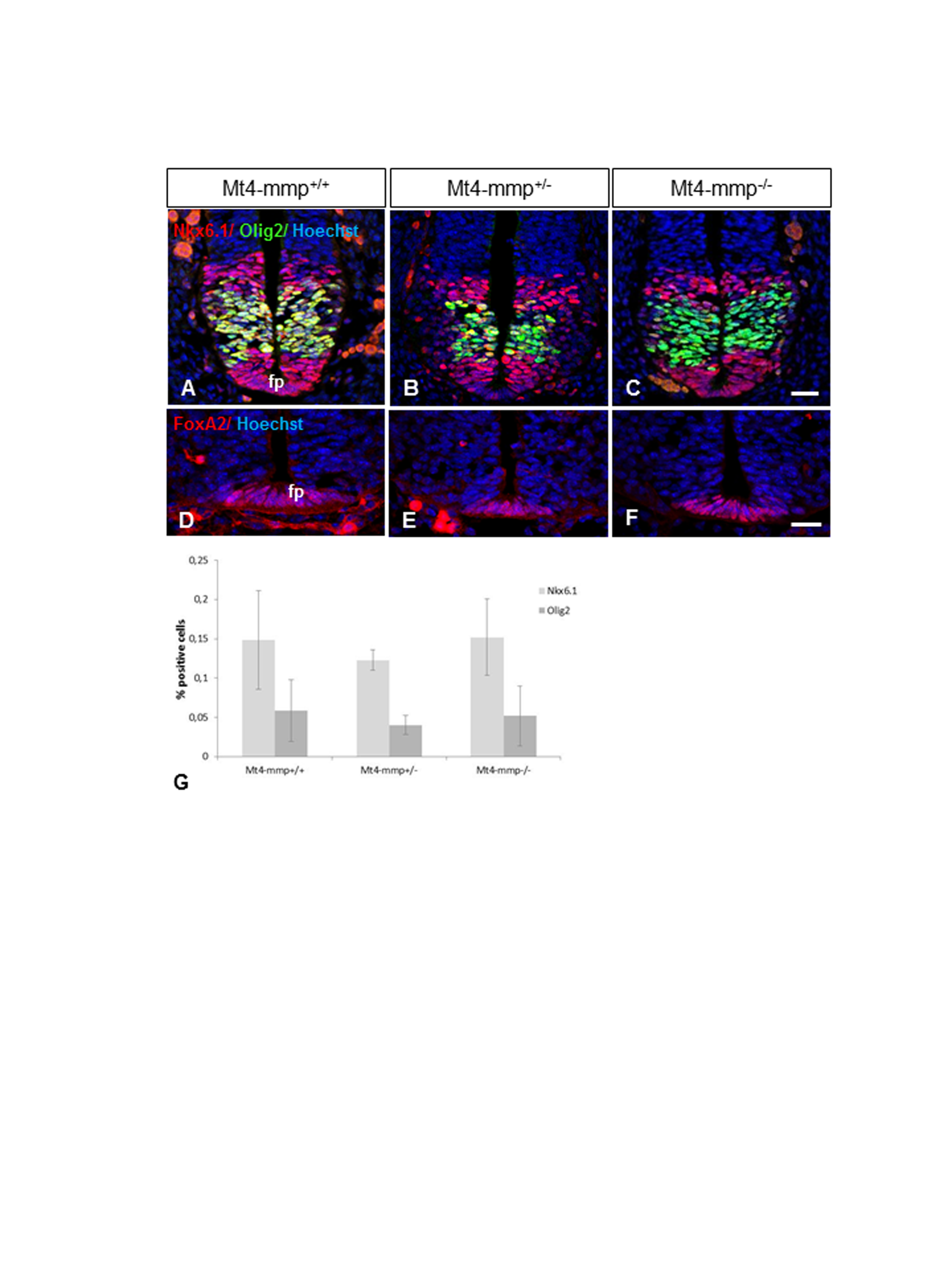

Supplement: S2 Fig — (A-C) Double-labelling immunohistochemistry for the transcription factors Nkx6.1 (red) and Olig2 (green) in the neural tube of E10.5 WT, HT and KO embryos. (D-F) Immunostaining for FoxA2 (red) demonstrated that the floor plate is properly specified in the WT, HT and KO neural tubes. Sections were incubated with Hoechst for nuclear staining (blue). (F) Quantification of the percentage of Nkx6.1 and Olig2-positive cells relative to the total area of the neural tube in the WT, HT and KO embryos. Data are presented as mean ± SD (n = 3 per each genotype). One-way ANOVA analysis revealed no statistical differences in the percentage of positive cells among the WT, HT and KO neural tubes. Abbreviations: fp, floor plate. Scale bars: 50 μm (A-C); 20 μm (D-F). (TIF) [file pone.0184767.s002.tif]
